# Is This Within Reach? Left but Not Right Brain Damage Affects Affordance Judgment Tendencies

**DOI:** 10.3389/fnhum.2020.531893

**Published:** 2021-01-27

**Authors:** Jennifer Randerath, Lisa Finkel, Cheryl Shigaki, Joe Burris, Ashish Nanda, Peter Hwang, Scott H. Frey

**Affiliations:** ^1^Department of Psychology, University of Konstanz, Konstanz, Germany; ^2^Lurija Institute for Rehabilitation Science and Health Research, Kliniken Schmieder, Allensbach, Germany; ^3^Department of Psychological Sciences, University of Missouri, Columbia, MO, United States; ^4^Department of Physical Medicine and Rehabilitation, University of Missouri, Columbia, MO, United States; ^5^Department of Neurology, University Hospital, Columbia, MO, United States; ^6^Stroke and Neurointerventional SSM Neurosciences, St. Clare Hospital, Fenton, MO, United States; ^7^Department of Neurology, Saint Louis University Hospital, St. Louis, MO, United States

**Keywords:** stroke, perception action, affordances, lesion analysis, reachability, decision making

## Abstract

The ability to judge accurately whether or not an action can be accomplished successfully is critical for selecting appropriate response options that enable adaptive behaviors. Such affordance judgments are thought to rely on the perceived fit between environmental properties and knowledge of one's current physical capabilities. Little, however, is currently known about the ability of individuals to judge their own affordances following a stroke, or about the underlying neural mechanisms involved. To address these issues, we employed a signal detection approach to investigate the impact of left or right hemisphere injuries on judgments of whether a visual object was located within reach while remaining still (i.e., reachability). Regarding perceptual sensitivity and accuracy in judging reachability, there were no significant group differences between healthy controls (*N* = 29), right brain damaged (RBD, *N* = 17) and left brain damaged stroke patients (LBD, *N* = 17). However, while healthy controls and RBD patients demonstrated a negative response criterion and thus overestimated their reach capability, LBD patients' average response criterion converged to zero, indicating no judgment tendency. Critically, the LBD group's judgment tendency pattern is consistent with previous findings in this same sample on an affordance judgment task that required estimating whether the hand can fit through apertures (Randerath et al., 2018). Lesion analysis suggests that this loss of judgment tendency may be associated with damage to the left insula, the left parietal and middle temporal lobe. Based on these results, we propose that damage to the left ventro-dorsal stream disrupts the retrieval and processing of a stable criterion, leading to stronger reliance on intact on-line body-perceptive processes computed within the preserved bilateral dorsal network.

## Highlights

- Patients suffering from stroke demonstrate task specific deficit profiles for affordance judgment skills.
- Effects of stroke on affordance judgment skills appear lesion specific.
- Structures in the left brain appear to be necessary to apply learned judgment tendencies when judging actor-related affordances.

## Introduction

Whether an action is suitable to be performed depends on reciprocity between properties of the environment and the subjects' capabilities (Shaw et al., 1982; Turvey, 1992). Accurately determining this is essential for action selection and subsequent adaptive behavior (Frey and Grafton, 2014). Gibson (1979) initially suggested that action is guided by the *perception of affordances*, in the sense that environmental properties directly offer information prompting for or *affording* certain actions. Thus, he termed the ability of perceiving action opportunities “*affordance perception*” (Gibson, 1977). Perceiving affordances is highly relevant in daily life pertaining, for example, to reaching or grasping objects, climbing stairs, walking through apertures or crossing a street. Thus, whilst navigating through our environment and interacting with objects, we are frequently confronted with the necessity to quickly decide whether we are capable to execute a particular action.

In scientific works on affordance perception, some authors focused on object and tool manipulation such as functional tool use or grasping [for a review see: Borghi and Riggio (2015); Sakreida et al. (2016); Osiurak et al. (2017)]. Others, such as the current study, instead focused on tasks (e.g., reaching or climbing) with the actor-related abilities representing a central aspect. These studies regard the individual subject as major reference, emphasizing that affordances are unique to individuals and need to be considered relative to their capabilities. A series of behavioral studies have shown that healthy young adults are primarily able to perform appropriate decisions in such actor-related affordance judgment tasks [e.g., judging the ability to step across obstacles (Cornus et al., 1999; Daviaux et al., 2014), or passing through apertures (Franchak et al., 2012; Randerath and Frey, 2016)]. Another frequently investigated ability involves judging whether objects are located within reach (i.e., Reachability) (Carello et al., 1989; Gabbard et al., 2005, 2006; Randerath and Frey, 2016). The literature suggests, that there is considerable variability in affordance judgment performance across tasks. For example, subjects may demonstrate overestimations (e.g., reachability task: Gagnon et al., 2013; Randerath and Frey, 2016) vs. underestimations (e.g., walking though doorways task: Davis et al., 2010; Hackney and Cinelli, 2011) when judging their capabilities.

Of relevance to individuals with acquired disabilities (e.g., as a result of stroke), several studies have demonstrated that healthy subjects are able to adapt their affordance judgments to altered body constraints. For instance, Pepping and Li (2000) showed that subjects made also accurate judgments when judging the maximum reachable height while wearing weights. Moreover, in a recent study we demonstrated that young and older adults can adjust their affordance judgments in a task that required determining whether or not their hands could pass through a variable sized opening (i.e., Aperture Task) after being equipped with a hand splint (Finkel et al., 2019b).

The variable results in affordance judgment performance across tasks in the literature, the ability of healthy persons to adapt performance to altered body constraints and also existing theoretical models (ACH; Cisek, 2007; Cisek and Kalaska, 2010), suggest that affordance judgments are based on a complex and flexible ability involving multiple sources of information. These include perception of environmental properties, specific task requirements, perception of own capabilities and/or experience due to practice. Accordingly, we expect a complex brain network to be engaged in affordance judgments, integrating attentional, perceptual, and motor cognitive processes.

Although it is reasonable to presume that brain damage due to stroke may affect actor-related affordance judgments due to sudden changes in motor or cognitive abilities or both, to our knowledge attempts to study these behaviors and their underlying mechanisms are rare. There is evidence for a correlation between errors in perceiving maximum reaching distance and risk for falling in hemiplegic patients (Takatori et al., 2009). Stroke patients who have more falls and more restricted walking mobility have also been shown to collide more frequently with doorways on the affected side of the body (Muroi et al., 2017). In prior work, we employed signal detection analyses and lesion analyses to explore actor-related affordance judgments based on the upper extremity and their neural substrates (Randerath et al., 2018). Compared to healthy controls, stroke patients experienced greater difficulty estimating whether their hand could fit through rectangular apertures of different sizes. In this Aperture Task, lower perceptual sensitivity went along with lesions in distributed bilateral brain mechanisms (claustrum, cingulum and ventro-dorsal fronto-parietal regions). In patients with left brain damage, errors were associated with lesions in sites typically implicated with impaired action planning and simulation processes (Buxbaum et al., 2007; Frey, 2007; Goldenberg, 2009; Randerath et al., 2009, 2010; Vingerhoets, 2014), i.e., limb apraxic behavior as demonstrated by difficulties with imitating hand gestures. In right brain damaged patients, errors were related to lesions in regions typically associated with perceptual impairments in visuo-spatial tasks (Mort et al., 2003; Golay et al., 2008; Karnath et al., 2011), i.e., visuo-spatial impairment as demonstrated by difficulties with line-bisection.

In the present study, we applied signal detection theory (Green and Swets, 1966; Macmillan and Creelman, 1991; Fox, 2004; SDT) to investigate effects of lateralized brain damage on reachability judgments. Likewise, we also employed voxel-lesion symptom mapping (VLSM) (Bates et al., 2003) and voxel-based subtraction analyses to explore possible underlying mechanisms in this same sample of participants.

Further, whether a target is actually located within reach is heavily influenced by body constraints (e.g., arm length, flexibility, coordination, stability, strength). It seems reasonable to assume that an altered body state (e.g., motor and flexibility limitations due to stroke related hemiparesis) influences the decision process. This may lead to increases in uncertainty or erroneous judgments. In the current study, participants were asked to judge whether they could reach an object, and respond “yes” or “no” accordingly. They were asked to remain still while determining whether an object presented at varying distances on one of three tracks (left, middle or right) was located within reach (Reachability Task). In addition to standard accuracy values, we used SDT to more thoroughly investigate the impact of stroke on affordance judgments. Specifically, we assessed variables of judgment tendency and perceptual sensitivity to comprehensively decipher judgment behavior. These variables provide important information on response quality including error characteristics. Due to our assumption, that perceptual processes are also involved in affordance judgments, the testing procedure included a control task (a perceptual estimation task, i.e., Depth Perception Task) in order to test whether perceptual abilities involved in depth or distance perception are correlated with judging the reachability of a presented object.

Based on our previous patient study using the Aperture Task (Randerath et al., 2018), we predicted that both patient groups would show worse performance compared to controls as a result of lowered perceptual sensitivity measures. More precisely, we expected significant correlations between perceptual sensitivity and apraxia scores (measured by hand imitation and pantomime of tool use) in the LBD group as well as significant correlations between self-evaluated motor capacities and all measured signal detection variables in both patient groups. In contrast to our previous study, no effects of neglect were expected, assuming that visuo-spatial components load less on the Reachability Task as compared to for example the Aperture Task.

Secondarily, based on previously reported results using a between-subjects design in healthy young adults (Randerath and Frey, 2016), we further predicted that, both, healthy participants and patients would show liberal judgment tendencies in the Reachability Task tending toward a higher rate of False Alarms and Hits.

Finally, we expected that lesion analyses would reveal bilateral ventro-dorsal as well as dorso-dorsal lesion sites being associated with lowered perceptual sensitivity (Randerath et al., 2018).

Our results are only partly in support of these hypotheses.

## General Methods

We implemented the Reachability Task in the same sample of participants as in our previously published study that tested subjects' ability to judge whether their hand can fit into a given aperture (Aperture Task; Randerath et al., 2018). To not interfere with therapy plans, all tasks were administered within two consecutive days at a weekend. Each day consisted of two sessions of ~45 min each. Per day participants solved one experimental and one neuropsychological session with at least 1-h break in between. Half of the group was randomly assigned to start with the Aperture paradigm on day one and performed the Reachability paradigm on day two, the other half started with the Reachability paradigm on day one and performed the Aperture paradigm on day two. The study was conducted at the RUSK Rehabilitation Center in Columbia, Missouri and approved by the University of Missouri—Columbia Health Sciences Institutional Review Board (HSIRB).

### Sample

A total of 64 individuals participated in all four sessions of the project. All of them fulfilled the inclusion criteria that were: right-handedness according to the Edinburgh Handedness Inventory (Oldfield, 1971), normal or corrected-to-normal vision (at least 30 f/9 m) and the declaration to have no (other) neurological or psychiatric diseases as well as the availability of the patients' brain scans. Healthy participants had to reach a score on the 3MS-R (revised version of the Mini Mental State Exam) that was larger than 88 (Tschanz et al., 2002; Alexopoulos et al., 2007). Furthermore, all participants were naïve to the specific goals and hypotheses of the study and gave their informed written consent in accordance with the local IRB and the Declaration of Helsinki. The study was performed in cooperation with the RUSK Rehabilitation Center in Columbia, Missouri, US. Demographic and clinical data are listed in Table 1.

**Table 1:** Demographic and clinical data.

	**Controls** ***N* = 29**	**LBD** ***N* = 17**	**RBD** ***N* = 17**
Gender: male/female	17/12	8/9	12/5
Age: mean (range)	61.69 (43–77)	62.47 (32–82)	64.47 (37-84)
Months since lesion onset: mean (range)	-	23.35 (0.25–116)	18.10 (0–134)
3MS-R-score: mean (range)	97.38 (91–100)	78.24 (52–97)	88.94 (71–98)
**Aphasia: impaired/not impaired**			
Read and obey (comprehension)	0/29	1/16	0/17
Naming body parts (production)	0/29	1/16	0/17
Neglect: yes/no			
Line Bisection	0/29	1/16	3/14
Star Cancellation	0/29	1/16	5/12
Motor function: mean (range)	5 (4.88–5)	3.04 (0–5)	2.43 (0–5)
**Apraxia: impaired/not impaired**			
Hand imitation	3/26	7/10	3/14
Pantomime	0/29	8/9	2/15
**Anosognosia: mean (range)**			
Self-evaluation	0.655 (0–5)	12.24 (1–23)	9.97 (0–22)
R-P-Discrepancy	−0.345 (−3–0)	0 (−5–8)	2.3 (−10–23)

#### Patients

Thirty-four patients with unilateral infarction (74%) or hemorrhagic stroke were identified as candidates for the current exploratory study by one of three attending physicians [A.N. (University of Missouri Hospital), J.B. and P.H. (RUSK Rehabilitation Center)]. The patient population was representative for a neurorehabilitation center treating acute, subacute and chronic stroke patients. Half of the patients had a left brain damage (LBD) and the other half suffered right brain damage (RBD). Brain scans indicated a prior stroke or additional small lesions for five patients (LBD02, LBD03, LBD28, RBD02, RBD25).

Due to the inclusion of patients with and without hemiparesis, all patients indicated their responses in the affordance judgment tasks using their unaffected ipsilesional hand.

#### Healthy Controls

Thirty healthy subjects participated in the study. They were recruited via advertisements. Healthy subjects were assigned to one of two control groups: a control group that used their left hand to indicate responses (CL, yoked with LBD patients) or a control group that used their right hand (CR, yoked with the RBD patients), respectively. Age and gender distribution were matched between patient and control groups. One participant in the CR group was excluded due to his low 3MS-R-score leading to 29 healthy participants.

### Neuropsychological Assessment and Motor Function Testing

All participants took part in a comprehensive neuropsychological assessment and were additionally tested in their motor function. Please note that we here only describe those neuropsychological tests that are relevant for our hypotheses and where there are performance differences between patient groups and healthy controls. Participant's motor-cognitive abilities were assessed by testing imitation of meaningless hand posture [Cut-off: 18 points, Goldenberg (1996)] and the production of tool-use pantomime gestures [Cut-off: 45 points, Goldenberg (2003)]. Motor function of the contralesional upper extremity was tested (Wolf Motor Function Test (WMFT), Wolf et al. (2001)]. We used a short test version including nine out of 17 tasks that involve gross arm and shoulder movements (forearm to table, forearm to box, hand to table, hand to box, extend elbow) and finer hand and finger movements (lift can, lift pencil, stack checkers, flip cards) [adapted Cut-off: mean of 3 in functional ability]. A possibly existing anosognosia for motor impairment was assessed using the VATA-M-questionnaire limited to upper arm motor abilities (Della Sala et al., 2009). Beside patient's self-evaluation of performing certain motor tasks, the estimation of an independent rater was acquired. The rater-patient discrepancy score provided information on whether there is a diagnosis of anosognosia [Cut-off: R-P discrepancy score above 3]. Visuo-spatial deficits were diagnosed by the subtests line bisection and star cancellation of the Behavioral Inattention Test (BIT) (Wilson et al., 1987). Language deficits have been identified by a language production task (naming body parts) and a comprehension task (read and obey) as part of the 3MS-R.

## Material and Procedures

### Material

**Figure 1:**
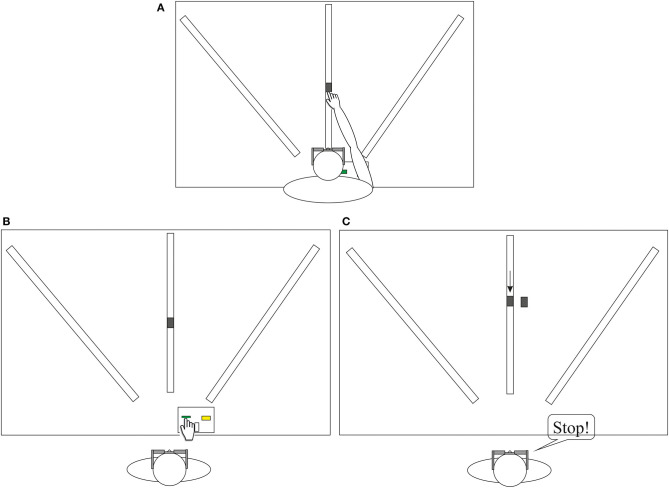
The figure depicts the measurement procedure **(A)**, the Reachability Task **(B)** and the control task including perceptual estimations **(C)**. Participants were seated centrally in front of the reaching apparatus. The example shows a setting for the right hand (RBD and CR group). **(A)** To determine the maximum reach participants successively pushed the object as far as possible with their index-finger along each of the three tracks, while goggles were closed. Bending forward was allowed but the bottom needed to stay seated. **(B)** Upon vision, participants were asked to respond as accurate as possible whether they judged the object to be reachable, by pressing a designated yes or no button. **(C)** The participants said stop, when they decided that the moving object was aligned with the object next to the track and were allowed to indicate final adjustments. The image depicts a trial in which the object was moved toward the participant.

The custom-made reachability apparatus consisted of a height adjustable table with three tracks mounted onto it. On each track one rectangular object was presented. These objects could be manually moved within the particular track. Object-distances were adjusted with the help of mounted measurement tapes. Participants were seated with a seatbelt around their hips to prevent them from moving their bottom from the seat. The table top was height-adjusted to the participant's solar plexus. The distance to the table was 25 cm. Participants wore plato-goggles throughout the experiment to control for visual feedback during measurements and adjustments (Figure 1). Wearing goggles throughout the experiment additionally allowed for response time measurements (see Supplementary Material).

### Measurements

The procedure started with measuring the maximum reachability (see Figure 1A). To determine the maximum reach of one assigned side the participants had to push each object with their index-finger along each track (left, middle, right) as far as possible, while goggles were closed. Bending forward was allowed but the bottom needed to stay seated. This procedure was repeated for three times and the maximum achieved value was used to determine the individual's increment settings for the experimental Reachability Task. The seatbelt and table edge prevented participants from losing their equilibrium while reaching forward.

### Affordance Judgments

Participants were not allowed to actually perform the movement, but only had to indicate decisions. In the Reachability Task there was one object presented at a set distance on one of three tracks: middle, left, or right (see Figure 1B). Upon the condition of staying seated, participants had to decide whether the presented object was within reach for the unaffected, ipsilesional hand or in healthy subjects for the assigned side, respectively. In accordance with task instructions, “within reach” meant that participants “can successfully touch the copper sensor on the object with the index finger” while staying seated. Participants were asked to indicate the decision by pressing the respective designated yes- or no-button on the response-device: “Press the green button on the left, when you think, “yes” I can reach the sensor with my index finger. Press the yellow button on the right, when you think, no you cannot reach the sensor with your index finger.” The presented distance was varied using fixed negative and positive increments allocated to the individual's maximum reachable distance (−16, −8, −4, −2, ±0, +2, +4, +8, +16 cm). Participants completed 54 trials (two repetitions × three tracks × nine increments). The 0 increment represented the maximal reachable distance for which the correct response would be “yes.” We added one filler trial per set of increments for which the correct answer would be “no” (further than +16 cm) to achieve the same amount of correct “yes” and “no” trials in a total of 60 trials. Judgments were solved for one side only, the same hand they indicated the responses via button press with (patients: unaffected ipsilesional hand; healthy participants: assigned left or right hand). The response box was positioned between two tracks. This was set either left or right from the center track depending on what hand pressed the buttons (ipsilesional). Shortly before goggles were opened, it was verbally indicated on which track the next object was presented (left, middle, or right). This supported the participants in orienting toward the correct side. One trial consisted of: (1) holding a neutral home-button pressed while goggles were closed (experimenter prepared setting), (2) opening of goggles, (3) The participant released the home-button to indicate the reachability of the presented object by either pressing the yes or no button, (4) Then the participant returned to the home-button and goggles closed. There was no feedback at any time about whether it was actually possible to reach the presented object, thereby preventing learning by haptic or visual feedback.

The affordance judgment experiment started with demonstration trials, presenting extreme trials, i.e., close or far distances on the left and right track. Correct responses to extreme trials verified task comprehension.

Perceptual estimation task (depth perception). Participants sat in front of the reaching apparatus. For the depth estimation task participants had to indicate “stop” (verbally or by use of gestures) as soon as a gradually adjusted object on the track was aligned with a rigid object next to the track, whereby final corrections were allowed. The rigid object was presented twice in two distances: + 16 and −16 cm from actual maximum reach to cover the range of distances. The movable object on the track was positioned either about 8 cm before or behind the fixed object. In total, 12 trials were presented with three tracks × two start-positions × two distances. Trials were presented in a fixed randomized order. The procedure is described in Figure 1C.

### Data Analyses

#### Analyzing Affordance Judgments

First, we ran a detailed analysis for affordance judgment performance in the Reachability Task, similar to the methods and the proceeding described in our previous studies that were used to analyze the Aperture Task (Randerath and Frey, 2016; Randerath et al., 2018). The following affordance judgment skills were analyzed: judgment accuracy (%) and the detection theory measures perceptual sensitivity (d-prime) and judgment tendency (c), that were both calculated on the basis of Hit and False-Alarm rates.

Tests of normality (Chi-Square-Test and screening of normal probability plots) showed that data have to be analyzed non-parametrically, since the Hit Rate (RBD, Controls) appeared not to be normally distributed (*Chi-Square* > 13.4, *p* < 0.041). Behavioral data were analyzed with SPSS 21 (IBM). In general, statistics are reported 2-tailed (*p* < 0.05). Whenever computing power was sufficient exact instead of asymptotic (*p*_*asymp*_) *p*-values were reported.

Overall group differences (controls, LBD patients, RBD patients) were assessed with the Kruskal Wallis Test.

Affordance judgments were analyzed by using the variables described below. Please note that we collapsed data across tracks in our analyses, since the comparison was not considered relevant for the formulated hypotheses. For completeness, Hit and False Alarm rates per group and track as well as *post-hoc* analysis are described in Supplementary Figure 1 and text. Additional information on response time measures is also provided in the Supplementary Table 1.

##### Judgment Accuracy (%)

A possible effect of group and task on judgment accuracy (percent of correct judgments) was analyzed.

##### Detection Theory Approach

We added detection theory variables to depict judgment behavior entirely. Judgment accuracy alone only provides information on percent values of accurate judgments but not on response quality including error characteristics (Miss: indicating “no” even when the object is within reach; False-Alarm: saying “yes” even when the presented object is not within reach). Thus, analyzing the response quality enabled us to better understand the processes that underlie judgment performance as well as the potential consequences of misjudgments. For example, the same level of accuracy can be achieved based on either rather risky or anxious-avoidant behavior, by using either more liberal (i.e., increased frequency of false alarms and hits) or more conservative (i.e., increased frequency of correct rejections and misses) judgment tendencies. Furthermore, because the detection theory measure for perceptual sensitivity is conceptually independent of the judgment tendency measure, the perceptual sensitivity parameter provides additional information on accuracy and participants' ability to discriminate between possible (“Hit”) and impossible actions (“Correct rejection”) (please see Macmillan and Creelman, 1991, p. 27, 41).

Theoretically, the basic assumption of SDT is that decisions are based on comparing observations with a criterion (Pastore and Scheirer, 1974). Following the detection theory approach, we calculated the following main variables of interest based on Hit and False-Alarm rates: subjects' perceptual sensitivity (discriminability index, d-prime), judgment tendency (criterion, c) and perceptual accuracy (area under the curve, AUC) (Green and Swets, 1966; Macmillan and Creelman, 1991; Fox, 2004; Brown and White, 2005). The False-Alarm Rate depicts the ratio of the number of negative events wrongly categorized as positive (False Alarm, i.e., indicating “yes” in trials, the presented object is not within reach) and the total number of actual negative events. The Hit Rate is calculated by the ratio of the number of positive events successfully categorized as positive (Hits; i.e., indicating “yes” in trials the presented object lies within reach) and the total number of actual positive events.

Judgment tendency was measured by the criterion (c): A conservative judgment tendency is reflected by a positive c-value (i.e., the subject responds “no” more often than the ideal observer), while negative c-values indicate a liberal judgment tendency (i.e., the subject responds “yes” more often than the ideal observer). The judgment tendency was calculated using the following formula: c = −0.5^*^[Z(Hit rate) + Z(False-Alarm rate)].

Perceptual sensitivity was measured by the discriminability index (d-prime): The more sensitive the participant is at discriminating (e.g., between objects within reach and objects out of reach), the larger the d′ value will be. As described in the literature, the discriminability index is independent of the criterion (Macmillan and Creelman, 1991; Fox, 2004). The perceptual sensitivity was calculated using the following formula: d′= Z (Hit rate)–Z(False-Alarm rate).

The perceptual or diagnostic accuracy is defined by the Area Under the Curve (AUC). Plots representing perfect discrimination pass through the coordinates 0 and 1, indicating 100% sensitivity *(Hit Rate, sensitivity)* and specificity *(False-Alarm Rate, 1-specificity)* and result in an AUC value of 1.

To correct for family-wise error rate, we additionally reported adjusted *p*-values using the stepwise Holm Bonferroni procedure (p_adj_) with *n* = 6 for the calculated accuracy and SDT variables.

Due to the multiplicity problem in such exploratory studies, we here also report adjusted *p*-values. However, as many other clinical studies with small patient populations, the present study has largely explorative character by collecting data also objective-driven and not only driven by pre-specified hypotheses. This makes *post-hoc* testing and a more flexible approach for design and analysis necessary. Being aware that multiple significance tests should only be used for descriptive purposes and not for definitive statements, we here base the interpretation and discussion of our results on both, adjusted and non-adjusted *p*-values (Bender and Lange, 2001; for a discussion and concluding advices, please see Armstrong, 2014).

#### Perceptual Estimation Task

Values were determined by the difference between actual measure and estimated values, i.e., in the depth perception task the difference between the fixed object and the moved object was determined (position in mm). To see whether there are group differences for depth perception a Kruskal Wallis analysis was run with the between-subjects-variable group (Controls, LBD patients, RBD patients). In addition, the correlation between deviations in depth perception and judgment accuracy (%) was analyzed (Kendall's Tau).

#### Neuropsychological Assessment and Affordance Judgments

In order to explore a potential correlation between neuropsychological symptoms after stroke and response behavior, we calculated correlations of affordance judgment skills with deficits in motor function, self-evaluation of motor abilities and anosognosia for motor function, motor cognition and visuo-spatial-processing (Kendall's Tau). To correct for family-wise error rate, we additionally reported adjusted *p*-values using the stepwise Holm Bonferroni procedure (p_adj_).

### Lesion Data Analysis

We applied standard voxel-wise lesion symptom mapping (VLSM) to determine damaged brain regions that are associated with deficient affordance judgment tendencies. We therefore semi-automatically delineated lesions from MRI or CT scans. Since recent evidence indicated that small sample sizes provide unstable results in VLSM (Lorca-Puls et al., 2018), we here additionally used a more conservative voxel-based subtraction analysis approach. The subtraction approach can be useful to distinguish the associated functional regions from those areas that are frequently damaged after unilateral stroke in general (Rorden and Karnath, 2004).

#### Lesion Delineation

Lesions were mapped and delineated with a semiautomatic approach using SPM 8 [Clusterize Toolbox, (Clas et al., 2012; de Haan et al., 2015)]. Afterwards brain and lesion maps of each patient were spatially normalized with SPM 8 [Clinical Toolbox, https://www.nitrc.org]. The spatial position of the resulting Volumes of Interest was subsequently checked by comparing the lesions on each individual's structural scan with the VOI displayed on the ch2-template distributed with MRIcron Software (Rorden and Brett, 2000). Where adjusting was required, lesion maps were manually corrected using MRIcron.

#### Statistical and Subtraction Analysis of Neural Correlates

The goal was to explore potential lesion correlates of a deviant judgment behavior in terms of judgment tendency (i.e., lack of stable response criterion,—a criterion that is close to zero) as the dependent variable. The individual direction of judgment tendencies (liberal or conservative) was ignored by converting all criterion scores into positive absolute values.

Statistical analysis was performed by using the non-parametric Brunner-Munzel test in NPM (Non-parametric Mapping available with the software MRIcron). Results were mapped on the ch2template (MRIcron). Due to the lack of power caused by the small patient samples, we here present uncorrected data instead of FDR-corrected values and additionally present results of a classic subtraction analysis. For the subtraction analysis we divided the patient group by the median of criterion magnitude and built an overlay for each group. Subsequently group overlays were subtracted from each other. Subtraction results were mapped onto the ch2 template. Finally, lesion-locus was determined by the AAL-template provided by MRIcron. The examiner was naïve to the clinical profiles of the patients at the time of lesion mapping.

## Results

### Neuropsychological Assessment

Overall, the evaluation of the neuropsychological assessments demonstrated typical clinical characteristics for each patient groups which will be detailed below.

#### LBD Group (N = 17)

Patients with LBD showed impairments in typical limb apraxia tests such as in performing hand imitations (41%) (Goldenberg, 1996) as well as in production of transitive pantomime gestures (47%) (Goldenberg et al., 2003). LBD patients further show lower self-evaluation scores in the VATA-M (Della Sala et al., 2009) that went along with impairments in upper extremity motor function assessed with the WMFT (53%). Only 12 percent demonstrated anosognosia for motor impairment in the VATA-M. In this patient group we detected hardly any difficulties with visuo-spatial tasks. In both the line bisection and star cancellation tasks only one patient demonstrated deficits.

#### RBD Group (N = 17)

Some patients with RBD showed mild impairments in motor-cognitive abilities such as hand imitation (18%) or transitive pantomime gestures (12%). Further, more than half of the group demonstrated motor impairments in their contralesional upper extremity (53%) that correlated with lower self-evaluation scores. Additionally, 24 percent of RBD patients were identified to demonstrate anosognosia for motor impairment. Furthermore, in this group there were patients demonstrating difficulties in visuo-spatial abilities assessed with line bisection (18%) and star cancellation (29%) tasks (Wilson et al., 1987).

### Performance in the Reachability Task

Contrary to the a priori predictions, judgment accuracy and perceptual sensitivity measures did not demonstrate any significant effects of stroke. However, left brain damage appeared to alter the strategy used to judge reachability: the LBD group's criterion values seem to indicate no judgment preferences in contrast to rather liberal judgments in healthy controls.

#### Affordance Judgments

We failed to detect any significant effects of stroke on accuracy of reachability judgments. Statistical comparisons with Kruskal-Wallis analysis revealed no main effect of group (Table 2).

**Table 2:** Descriptive data for patient groups and age-matched controls and analyses of group effects (Kruskal-Wallis Test) in the Reachability Task.

	**LBD**	**RBD**	**Controls**	**Group effects**		
**Variable**	***Mdn***	***Mdn***	***Mdn***	***H*(2)**	***p_***asymp*.**_***	***p_***adj***_***
Judgment accuracy (%)	74.07	66.67	74.07	2.892	0.235	0.47
Perceptual sensitivity (d')	1.39	1.25	1.72	3.362	0.186	0.558
Criterion (c)	−0.09	−1.08	−1.04	8.199	0.017	0.085
False-alarm rate	0.29	0.63	0.54	5.909	0.052	0.208
Hit rate	0.77	0.93	0.98	11.739	0.003	0.018
Diagnostic accuracy (AUC)	0.73	0.65	0.70	1.738	0.419	0.419

*LBD, patients with left brain damage; RBD, patients with right brain damage; Mdn, median; p_asymp_, asymptotic p-values; p_adj_, adjusted p-values (Holm Bonferroni procedure, k = 6)*.

Further, in contrast to our predictions the current study did not demonstrate a significant difference between stroke patients and healthy controls in their discriminability index. Only the descriptive levels suggest slightly lowered perceptual sensitivity in patient groups when determining whether an object is within or out of reach.

However, statistical comparisons revealed (marginally) significant group differences in judgment tendencies. As hypothesized, pairwise group comparisons with Mann-Whitney U-Test indeed revealed that healthy controls did not differ from the RBD patient group (*U* = 206.0, *p* = 0.362, p_*adj*_ = 0.362) by applying a liberal judgment tendency. But in contrast to our hypotheses, LBD patients (*U* = 116.0, *p* = 0.002, p_*adj*_ = 0.011) significantly differ in their judgment tendency from healthy controls. While on average there was no obviously biased judgment tendency detectable in the group of LBD patients (neither toward underestimation nor toward overestimation), the groups of RBD patients and healthy controls considerably overestimated their reaching ability.

**Figure 2:**
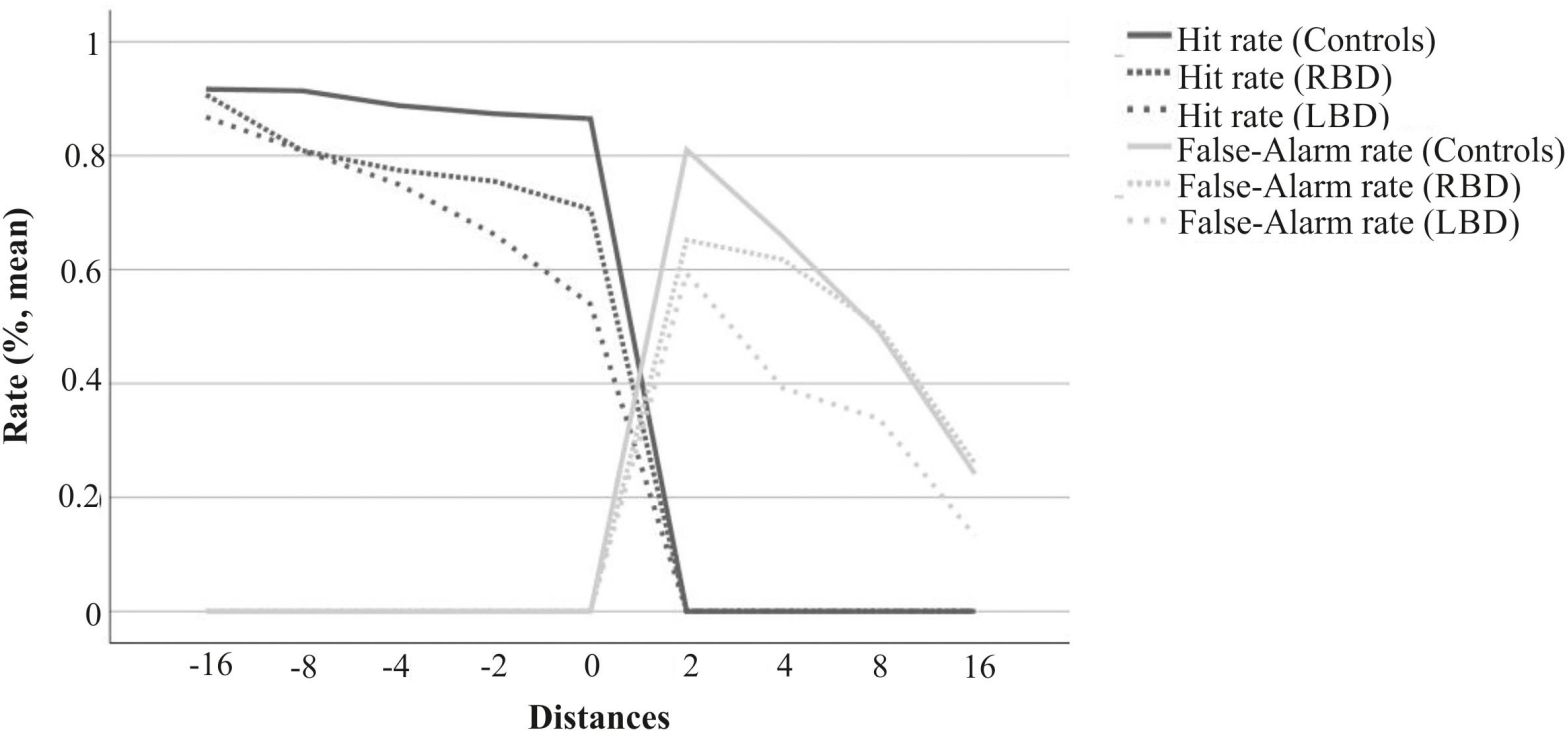
Hit and False-Alarm rates for LBD patients, RBD patients and age-matched healthy controls. The figure displays an overview of changes in Hit- and False-Alarm rates for the different distances across tracks. The value “0” reflects a trial with an object presented at the individually-defined maximum reachable distance. Distance-values represent deviations (cm) from the individual's maximum reachable target. Objects presented at positions with negative distance-values were located within the participant's actual reach (correct response: yes), and those with positive distance-values were located out of reach (correct response: no). The graphs display a typical distribution with higher Hit and lower False-Alarm rates for distances that are considerable further away from the physical constraints. Conversely, judgment performance for distances closer to the maximum reach (0) decreased.

More detailed information on response behavior is provided by the separate evaluation of Hit and False-Alarm Rate distributions (Figure 2). Further, Kruskal-Wallis test also revealed significant group differences for Hit rates. The healthy control group exhibited a close to perfect Hit Rate. Compared to the control group, the LBD group demonstrated a lower rate of Hits (*U* = 103.0, *p* < 0.001, p_*adj*_ = 0.002), whereas the RBD group showed a rather similar Hit Rate (*U* = 171.5, *p* = 0.065, p_*adj*_ = 0.261). Patient groups did not differ in judgment tendencies (*U* = 103.0, *p* = 0.157, p_*adj*_ = 0.471) nor in Hit Rates (*U* = 111.5, *p* = 0.254, p_*adj*_ = 0.508). This arises primarily from a considerable level of variance in patient groups' data. At least on a descriptive level, there seems to be a difference between LBD and RBD patients considering median values (see Table 2).

#### Perceptual Estimation

The maximum misjudgment per individual when estimating the alignment of two objects in distance was 0.51 cm in healthy controls, smaller than both the 1.19 cm in the LBD group and the 1.06 cm in the RBD group. Groups differed significantly in their estimations [*H*_(2_) = 15.96, *p* < 0.001]. Both patient groups estimated depth significantly worse compared to healthy controls (*U* ≥ 80.00, *p* ≤ 0.010). Group comparisons revealed no significant differences in depth estimation ability between patient groups (*U* = 110.5, *p* = 0.263). Thus, despite group differences in the perception control task, depth appeared to be perceived quite well with <1.2 cm deviation, which is within the shortest margin of increments (2 cm) presented for reachability judgments. For none of the groups, correlations between depth estimations and detection variables determining reachability judgments reached significance, which is in line with a previous report involving healthy young adults by Randerath and Frey (2016).

#### Correlations of Neuropsychological Assessment and Reachability Judgments

Driven by the formulated hypotheses, in LBD patients we calculated correlations between perceptual sensitivity and performance in apraxia tasks as well as the VATA self-evaluation score. To correct for family-wise error rate, we applied a stepwise Holm-Bonferroni correction (*n* = 7). In the RBD patient group, we calculated correlations between all applied reachability measures and self-evaluation of motor capacity (VATA-M) (Holm-Bonferroni correction, *n* = 5). Please note, that further exploratory analyses including all calculated correlations are presented in Supplementary Table 2.

In LBD patients, perceptual sensitivity in reachability judgments was not correlated with apraxia scores (hand imitation: *r* = −0.008, *p* = 0.967, *p*_*adj*_ = 0.967; pantomime: *r* = 0.105, *p* = 0.562, *p*_*adj*_ = > 1), but with the VATA self-evaluation score (*r* = −0.457, *p* = 0.012, p_*adj*_ = 0.084). The other judgment performance parameters were not significantly correlated with the VATA self-evaluation score (*r* ≥ −0.279, *p* ≥ 0.132, *p*_*adj*_ ≥ 0.264). The better patients estimated their own motor function to be, the more perceptual sensitive they displayed for judging reachability.

**Figure 3:**
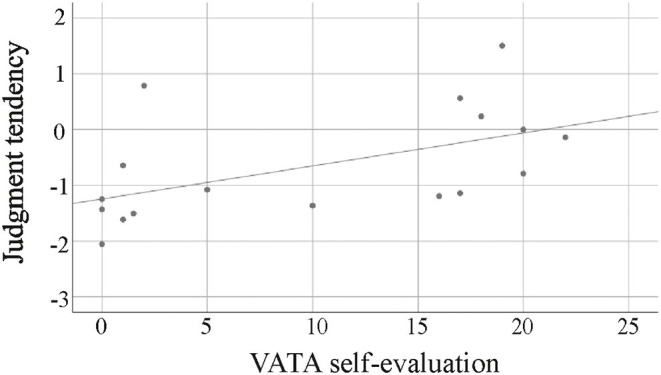
Judgment tendencies in RBD patients in relation to scores of self-estimated motor abilities (0: no problem to 3: problem). The worse RBD patients estimated their own bodily capabilities the more their criterion deviated from typical tendencies made by healthy subjects in the reachability task, converging to a 0-criterion.

In contrast, in the RBD patient group, VATA self-evaluation scores went along with almost all performance parameters in the Reachability Task (Hit Rate: *r* = −0.396, *p* = 0.038, *p*_*adj*_ = 0.076, False-Alarm Rate: *r* = −0.479, *p* = 0.009, *p*_*adj*_ = 0.036, Perceptual Sensitivity: *r* = 0.309, *p* = 0.089, *p*_*adj*_ = 0.089; Criterion: *r* = 0.466, *p* = 0.010, *p*_*adj*_ = 0.03, AUC: *r* = −0.481, *p* = 0.008, *p*_*adj*_ = 0.04). Correlations between self-evaluation scores and criterion are depicted in Figure 3. The better the patients thought their motor abilities to be, the more liberal their reachability judgments were; the more impaired patients estimated themselves to be, the less liberal their reachability judgments were.

Please note that additional correlation plots of VATA self-evaluation scores and dependent variables describing reachability judgments are attached to the Supplementary Figure 2.

### Lesion Data Analysis

**Figure 4:**
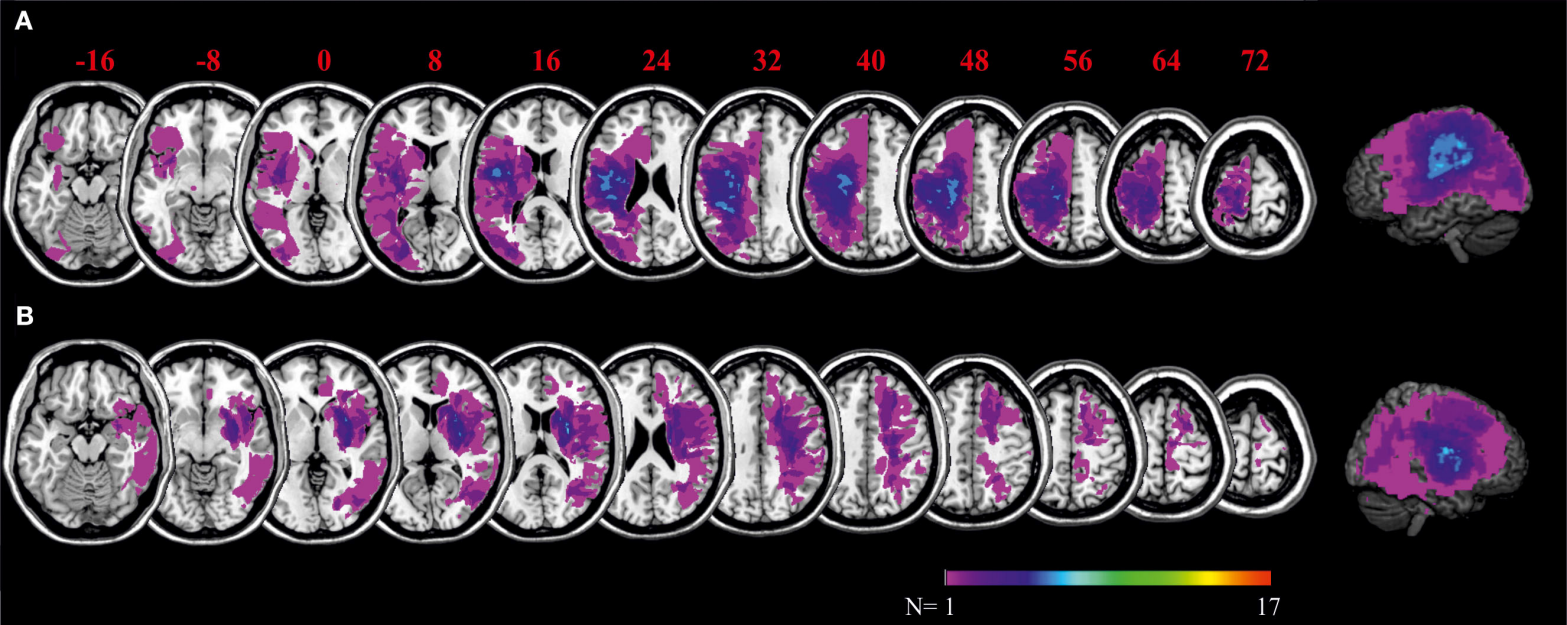
Overlays of LBD patients' **(A)** and RBD patients' **(B)** lesion maps. The color bar indicates degree of overlap of lesions out of 17 patients. MNI coordinates of each transverse section are given.

Figure 4 shows the distribution of lesions across the samples of left and right brain damaged patients, respectively. Lesions reached the margins of the middle cerebral artery territory. Maximum overlap of lesions was located in the center of the peri-sylvian region.

Behavioral results revealed that in contrast to the healthy control and RBD group, LBD patients applied no task-specific response strategy when judging affordances, meaning on group level, LBD patients demonstrated judgment tendencies with a close to zero criterion in the Reachability Task.

**Figure 5:**
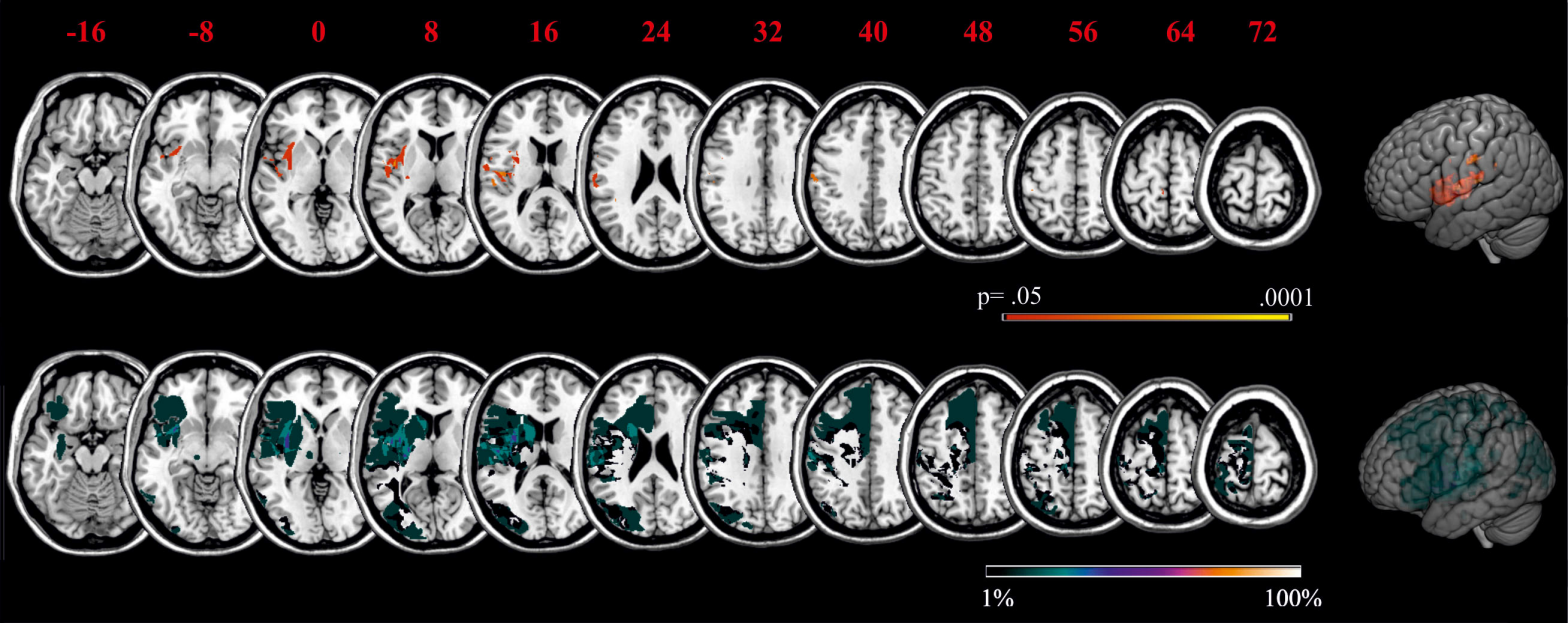
VLSM for the Reachability Task as well as subtraction analyses of the criterion in the Reachability Task. Statistical maps (upper graph) display voxels corresponding to a criterion close to zero, thereby stressing lesion sites of patients that had chosen no judgment tendency in the Reachability Task. Please note that in NPM analysis, only voxels were considered that were damaged in more than 10% of patients. Subtraction plots (lower graph) display voxels corresponding to a criterion close to zero, thereby stressing lesion sites of patients that had chosen no judgment tendency compared to patients that demonstrated a specific judgment tendency in the Reachability Task. Regions that were associated with a deviation from typical judgment tendencies were mainly located within the left insular, the left inferior parietal and the left middle superior temporal lobe. MNI coordinates of each transverse section are given.

In order to better explore potential underlying neuronal correlates for behavior of the LBD patient group, we computed a statistical VLSM analysis as well as a more conservative subtraction analysis with judgment tendency in the affordance tasks as the dependent variable. Using MRIcron software, brain regions were identified that were associated with low criterion magnitudes in the Reachability Task (Figure 5, upper graph). Because judgment tendency was not correlated with lesion volume (*r* = −0.258, *p* = 0.149), lesion volume was not considered as covariate in the current lesion analysis.

The statistical maps and subtraction plots for the Reachability Task demonstrated that ventral premotor areas (including the inferior frontal gyrus and adjacent striatum, claustrum and insula), as well as postcentral regions and adjacent inferior parietal areas with supramarginal gyrus were more frequently lesioned in patients that exhibited no judgment tendency (i.e., criterion close to zero) as opposed to patients showing a task-specific judgment tendency or bias.

## General Discussion

In the current study, we aimed to assess the effects of unilateral stroke on the ability to judge whether or not an object was located within reach. Based on previous results in a different affordance judgment task performed by the same participants, we expected significantly worse perceptual sensitivity in patient groups compared to healthy control subjects. However, neither perceptual sensitivity nor accuracy values differed between groups. Instead, the study revealed low magnitudes of judgment tendencies in the patient group with LBD compared to healthy controls and patients with RBD.

First, our main results indicate that stroke can affect affordance judgments for the reachability of objects. In particular, lesions in the left hemisphere appear to affect judgment tendencies in the Reachability Task. Second, correlational analyses suggest that in LBD patients with deficiencies in building or retrieving a criterion, affordance judgments may then be predominantly solved by relying on perceptive processes involved in actual body estimates (i.e., self-estimated motor function when judging whether an object is within reach). Third, our exploratory lesion analyses suggested the integrity of left insular and ventro-dorsal structures to be necessary to build or to refer to a stable criterion for affordance judgment tendencies. Our initial findings described here seem to be compatible with results described in the literature on motor-cognitive and decision-making performance. Below we consider these main findings in greater detail.

**Figure 6:**
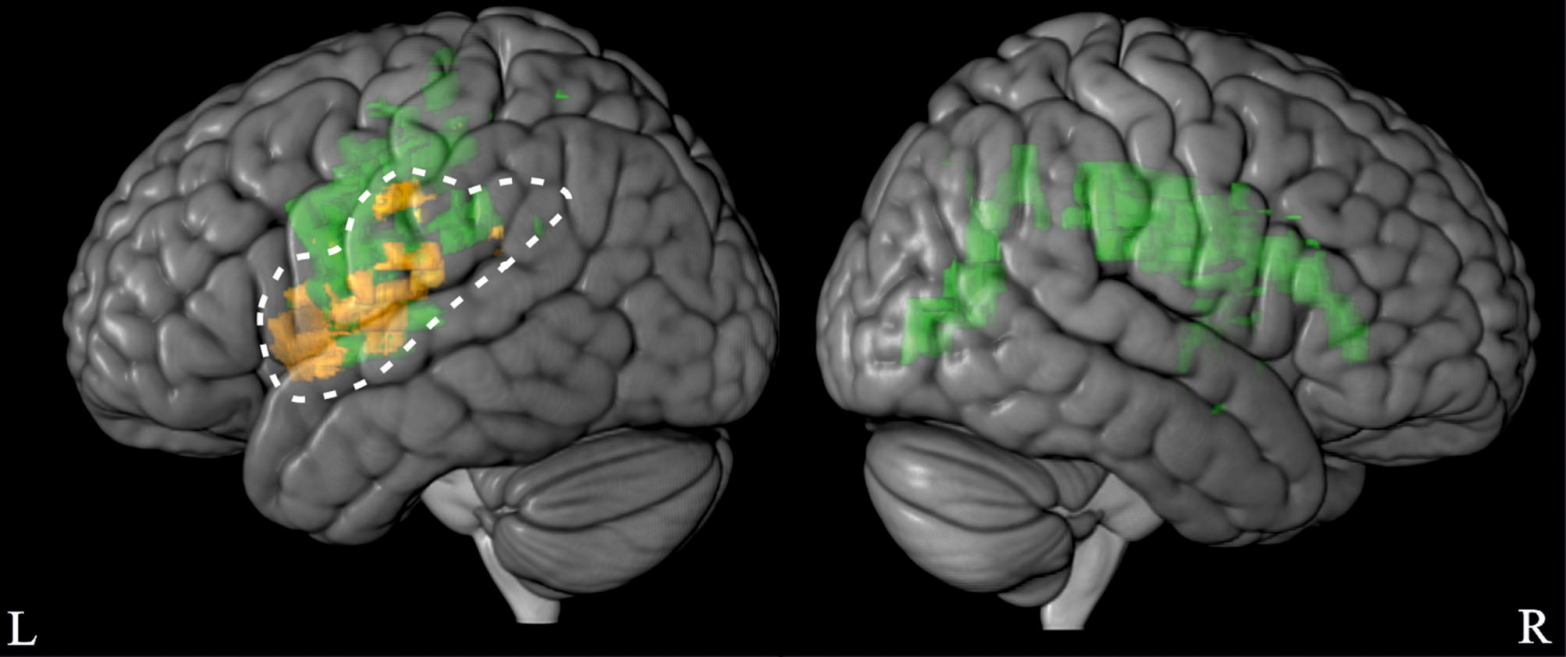
Working model providing first indications for essential regions for perceptual sensitivity and judgment tendencies in affordance judgments. In the light of the discussed results, we here propose a working model for essential elements when performing affordance judgments. For illustration purposes only, we here used the visually more clearly depicted VLSM results. In our working model, essential regions for judgment tendencies are depicted in orange (based on the Reachability Task, Figure 5) and those for perceptual sensitivity are displayed in green [based on the Aperture Task, (Randerath et al., 2018)]. The left hemisphere dominance for judgment tendency is highlighted.

Notwithstanding, the discussed issues clearly need further hypothesis-driven investigation. Future studies should consider testing larger samples, different types of affordance judgment tasks and if possible, include functional imaging. Here we will discuss a working model (Figure 6) that may aid further hypotheses driven testing. To this end, we also consider results of our previous study implementing the Aperture Task in the same stroke sample (Randerath et al., 2018).

Considering the current findings as well as results from previous affordance studies, affordance judgment performance appears to be highly dependent on task type and consequently on the involved relevant cognitive processes (e.g., perceptual abilities, prior experience and anticipated consequences, including risk perception or risk-taking behavior).

### The Left Hemisphere and the Criterion

Contrary to our *a priori* hypothesis, the Reachability Task demonstrated no group differences for accuracy nor for perceptual sensitivity measures. Instead, we found significant deviations in judgment tendencies after LBD. While healthy controls and patients with RBD overestimated the reachability of objects, LBD patients appeared to lack such a judgment tendency. This is consistent with our previous findings in this sample performing an affordance judgment task that required estimating whether the hand can fit through apertures (Randerath et al., 2018). For the so called Aperture Task healthy controls and patients with RBD demonstrated rather conservative judgment tendencies, which appears rather typical for this age group solving the Aperture Task (Finkel et al., 2019a). However, again for LBD patients such task specific judgment tendencies seemed to be absent.

At least two explanations seem possible. First, patients with LBD may demonstrate a general lack of utilizing any informed criterion, which might result from the known impairment of integrating different information in patients with left parietal lesions (Sirigu et al., 1995; Buxbaum et al., 2005). However, thus far lesion studies on risky emotional decision-making in patients with stroke did not demonstrate particularly lateralized results. And in contrast to our results, in this type of work typical lesion sites were rather anterior, i.e., ventromedial prefrontal cortex. But overlapping evidence seems to converge on one region, the insula (Clark et al., 2008; Weller et al., 2009). In comparison, statistical and subtraction analyses in our affordance judgment task demonstrated less ventromedial frontal but more posterior regions associated with a lack of response strategy. Aside from left insula lesions, these analyses emphasize left pre- and postcentral, superior temporal as well as inferior parietal lesion sites, known to be substantially involved in motor-cognitive abilities. Thus, perhaps the lack of evidence for use of a criterion affecting judgment tendencies predominantly seen in LBD patients may not be explained by a general lack of retrieving any criterion, but rather their being bound to motor-cognitive representations in actor-related affordance decisions. Perception of body-spatial metrics may remain intact in LBD patients, because of the involvement of a bilateral network (- probable more extensive in the right hemisphere) that is associated with processing spatial information (Mennemeier et al., 1997; Fink et al., 2000; Ciçek et al., 2009). While speculative, it is conceivable that patients with LBD lack a stable criterion bound to body structural representations and instead rely on the use of a flexible judgment strategy using current perceptual information via preserved brain regions. In line with this argument, our behavioral correlative analysis demonstrated associations of performance in perceptual sensitivity with measures that involve the actual estimation of bodily constraints. In LBD patients self-estimated motor function correlated with performing reachability judgments, and their hand size estimation correlated with performing judgments about whether their hand fits into a presented opening.

### Brain Damage and Task Specific Effects

Results appear to differ dependent on the applied task. In the current study the Reachability Task did not reveal any significant differences in perceptual sensitivity between patient groups and healthy controls. However, in our previously reported Aperture Task (Randerath et al., 2018) LBD and RBD patients both exhibited significantly greater difficulties in perceptual sensitivity when discriminating their hand's fit from a non-fit as compared to healthy controls. Further, healthy elderly controls and RBD patients applied a rather conservative judgment criterion when deciding whether their hand fits into an opening (underestimation), but a liberal judgment tendency when deciding whether an object is within reach (overestimation). Similarly, the choice of a differential criterion for judgment tendencies in the Aperture vs. Reachability Task has already been described by Randerath and Frey (2016) in a between-subjects design involving healthy adults. Considering these prior studies, the current study results are consistent with the idea that affordance judgments are dynamic, and that affordance judgment performance depends on task specific abilities.

In line with Gibson's ecological approach (Gibson, 1979) and more recent neural models [ACH: Cisek (2007); Cisek and Kalaska (2010); framework of the parieto-premotor cortical network for bodily self-recognition: Murata et al. (2016)], we propose that essential functional components in the affordance system may experience a heavier processing load compared to other system components that load less on the specific action task. When judging whether a part of the body fits into an opening it is feasible that visuo-spatial perception as well as knowledge about the dimensions of own body parts (e.g., hand size, shoulder width) are important. For judging the maximum reachability, it seems obvious that the knowledge about body dimensions (e.g., arm length) and the estimation of bodily capabilities (e.g., flexibility for bending forward, strength and stability) play an important role.

Brain damage due to unilateral stroke and related neuropsychological deficits may contribute to unraveling the complex interplay of variables involved in affordance judgments. Indeed, our study results provide some evidence that stroke and well-known related functional deficits appear to affect affordance judgments depending on the type of affordance judgment task (see working model in Figure 6). There is ample evidence that ventro-dorsal lesions in right brain damage frequently lead to visuo-spatial deficits (Ferber and Karnath, 2001; Karnath et al., 2011; Kerkhoff and Schenk, 2012). In fact, our previous study using the Aperture Task demonstrated significantly worse performance in perceptual sensitivity and clear associations between perceptual sensitive judgments and visuo-spatial abilities in RBD patients (Randerath et al., 2018). However, while visuo-spatial deficits after right brain damage seem to go along with the ability to sensitively dissociate between a hand's fit or non-fit into a horizontal opening, this functional deficit did not correlate with perceptual sensitivity scores in judging reachability. Instead, judgment performance in the Reachability Task seems to correspond to the perception of bodily capabilities. In the Reachability Task, the RBD patient group and healthy controls did not differ in perceptual sensitivity scores and exhibited a similar judgment pattern. Both the RBD patient group and healthy controls demonstrated overestimation of reaching abilities (liberal criterion). Further, RBD patients who estimated their bodily capabilities to be unaffected were particularly prone to overestimate their reaching abilities. In these patients, the non-consideration or misestimation of actual bodily limits or impairments might heighten the risk for fall or injury. By contrast, RBD patients who perceived their bodily capabilities to be affected by hemiparesis (measured by the VATA-M self-evaluation score), adjusted their criterion toward zero. It appears that an altered body state due to restrictions in the contralesional upper extremity may go along with a heightened uncertainty in affordance judgments. Interestingly, the patients' actual performances on the Wolf Motor Function Test were not correlated with performance in the Reachability Task. These findings corresponded with an earlier study in healthy adults by Gabbard et al. (2007) who showed that a more conservative reachability judgment strategy is attributed to greater perceived postural demands.

### Working Model

As depicted in Figure 5 and in our working model in Figure 6, our lesion analysis indicated that patients who seem to generate an atypical decision strategy often suffered from left brain damage in ventro-dorsal regions. These lesion locations were found to possibly contribute to the non-reliance on a stable criterion (i.e., “close-to-zero-tendency”). Many studies in the past century have demonstrated that damage to left ventro-dorsal regions frequently go along with motor-cognitive disabilities (Buxbaum et al., 2005; Rumiati et al., 2005; Goldenberg and Randerath, 2015; Weiss et al., 2016; Finkel et al., 2018). Left ventro-dorsal brain regions have been implicated in integrating perceptual information on body and object properties into an action plan (e.g., Buxbaum and Randerath, 2018).

The ventro-dorsal stream links temporal regions with the inferior parietal lobe and is known to be responsible for space perception, body part coding and semantically related object interaction (Rizzolatti and Matelli, 2003; Binkofski and Buxbaum, 2013) whereby the latter two functions appear to be predominantly lateralized to the left hemisphere (Schwoebel and Coslett, 2005; Goldenberg, 2009; Buxbaum and Randerath, 2018). In line with our results, other studies showed that insular lesions have been associated with an altered decision-making pattern in stroke patients compared to healthy controls [risky decision-making task (Cup Task) that separates risky gains and risky losses; Weller et al., 2009] and a reduced adjustment in a decision-making task including risk seeking behavior (Cambridge Gambling Task; Clark et al., 2008). The anterior insular cortex has been suggested to be a key structure in risk-taking behavior and to play a decisive role in the integration of sensory information into cognitive processes (Smith et al., 2014). These reports are consistent with our results, and it seems plausible that lesions in the insular cortex lead to a deviant response strategy in affordance tasks. That risk-taking behavior and associated therewith, anticipated potential consequences of misjudgments, can play a significant role in affordance judgment tasks, has been demonstrated by a previous behavioral study with young and older adults (Finkel et al., 2019a). It has been shown, that especially older adults were more conservative in their judgments and were also less likely to engage in domain-specific risky activities.

## Conclusions and Future Directions

Overall, our present exploratory results support the idea that the study of affordance judgments in patients with functional deficits may help to unravel underlying mechanisms of affordance judgments and characteristic resilience of these mechanisms. Processing or referring to a well-established criterion for actor-related affordance judgments appears attributable to a left lateralized network in the brain, possibly involving the ventro-dorsal pathway. Furthermore, the results of our previous studies support the idea that affordance judgments depend on a flexible and dynamic brain network which is highly task-specific. And, despite its potential relevance to daily functioning, this issue has been rarely addressed in patient populations. Even in healthy samples, relatively little is known about the actual effects of task specificity within subjects, since only a few studies have implemented more than one actor-related affordance judgment task in within subject designs (e.g., Pepping and Li, 2005; Weast et al., 2011; Franchak et al., 2012; Cole et al., 2013; Comalli et al., 2013; Day et al., 2015). Despite the small sample size, the current exploratory results may serve as a promising initial start fueling further investigations. Consequently, future research needs to replicate and extend the current results to improve our understanding of the affordance judgment concept. We propose that studying actor-related affordance judgments in patients with functional deficits as well as functional imaging studies with healthy subjects may help to gain a comprehensive understanding of the behavioral and neural mechanisms that underly affordance judgments. Particularly, the usage of SDT parameters has merits as it allows a more precise interpretation of participants' affordance judgment behavior. Applying the SDT approach in neuroimaging studies may potentially benefit the differentiation between involved perception, cognition and action modules or loops as proposed in recent neurophysiological models by for example Cisek and colleagues (Cisek and Kalaska, 2010; Pezzulo and Cisek, 2016). In addition, the trainability of such affordance judgments needs to be illuminated to test whether training may be beneficial for improving or compensating deficient performance caused by brain damage or sudden bodily alterations (e.g., caused by injury). Our daily life requires flexible adaptation to many different tasks, ranging for example from reaching for a coffee-mug on a kitchen table to crossing busy streets. Thus, in the long run, combining different affordance judgment tasks may potentially be useful for developing integrative motor-cognitive training strategies that might facilitate flexible judgments.

## Data Availability Statement

All datasets generated for this study are included in the article/Supplementary Material.

## Ethics Statement

The studies involving human participants were reviewed and approved by Health Sciences IRB University of Missouri-Columbia. The patients/participants provided their written informed consent to participate in this study.

## Author Contributions

JR and LF contributed equally to the manuscript. SHF and JR designed the work. JR, AN, CS, JB, and PH were responsible for data acquisition. JR and LF analyzed and interpreted the data. LF, JR, and SHF drafted the manuscript. All authors revised the manuscript.

## Conflict of Interest

The authors declare that the research was conducted in the absence of any commercial or financial relationships that could be construed as a potential conflict of interest.
