# Supplementary material for: Is This Within Reach? Left but Not Right Brain Damage Affects Affordance Judgment Tendencies
Source: Front Hum Neurosci. 2021 Jan 27;14:531893. doi: 10.3389/fnhum.2020.531893 (PMC7873490; doi:10.3389/fnhum.2020.531893)
Supplement: Supplementary file 1 [file Table_1.docx]

**Supplementary Material**

First, we here provide additional descriptive and statistical information (group comparisons applying Mann-Whitney U Test) on response time measures per group. Time measures represent the time between opening of goggles and releasing the start button for indicating judgment per button press. Importantly, participants’ task was to judge as soon as the goggles were opened, but also to answer as accurate as possible. Typically, participants responded slower in critical trials with distances close to the actual individual reaching distance and faster response times have been reported for more distinct trials with rather obvious deviations from actual reaching distance (Randerath & Frey, 2016).

Second, we provide supplementary graphs depicting the distributions of Hit and False Alarm rates per track.

Third, correlations between VATA self-evaluation score and reachability judgment variables are displayed in graphs. Furthermore, correlation results of additional and exploratory analyses are listed in a table without testing for multiple comparisons.

**Response times measures.**

| Supplementary Table 1.  *Response time data for patient groups and age-matched controls as well as group comparison results (Mann-Whitney U Test) in the Reachability Task.* | | | | | | | | | | |  |
| --- | --- | --- | --- | --- | --- | --- | --- | --- | --- | --- | --- |
| Variable | Descriptive data | | |  | Group comparisons | | | | | | |
|  | LBD | RBD | C |  | C vs. LBD | | C vs. RBD | | LBD vs. RBD | | |
|  | *Mdn* | *Mdn* | *Mdn* |  | *U* | *p* | *U* | *p* | *U* | *p* | |
| Response Time (ms) | 1689.56 | 1952.84 | 1137.90 |  | 113.0 | .004 | 128.0 | .006 | 126.0 | .736 |  |
| *Note.* For one LBD patient, response times cannot be measured. Kruskal-Wallis Test showed that groups differed significantly in their response times (*H*(2) = 11.241, *p* = .004). LBD = patients with left brain damage, RBD = patients with right brain damage, C = Healthy Controls | | | | | | | | | | |  |

**Effects of tracks.**

Friedmann-Test revealed that healthy subjects and RBD patients showed differential judgment accuracy depending on the track (Controls: *χ²*(2) = 6.02, *p* = .049, RBD: *χ²*(2) = 7.54, *p* = .021), while LBD patients demonstrated no differential judgment accuracy depending on the track (*χ²*(2) = 0.10, *p* = .969). On average, healthy controls demonstrated better performance when judging the left track (*M* = 74.90, *SD* = 10.77) compared to the middle track (*M* = 70.88, *SD* = 8.74; *Z* = -2.188; *p* = .027). RBD patients demonstrated best performance for the middle track (*M* = 71.90, *SD* = 12.80 *Z* ≤ -1.922 *p* ≤ .056), whilst there were no differences between performances for the other two tracks (left: *M* = 65.67, *SD* = 10.80; right: *M* = 66.99, *SD* = 10.29; *Z* = -0.354, *p* = .755). However, considering Hit and False Alarm rates, there was no main effect of track in the respective study group (Hit rate: *χ²*(2) ≥ 0.46, *p* ≥ .446; False Alarm rate: *χ²*(2)≥= 1.56, *p* ≥ .108). Thus, in contrast to accuracy values that were slightly modulated by the spatial location of tracks, the judgment performance measured with signal detection variables (Hit and False Alarm rate) is not considerably modulated by track across groups (see Supplementary Figure 1). Please note that p-values presented here are not corrected for multiple comparisons.


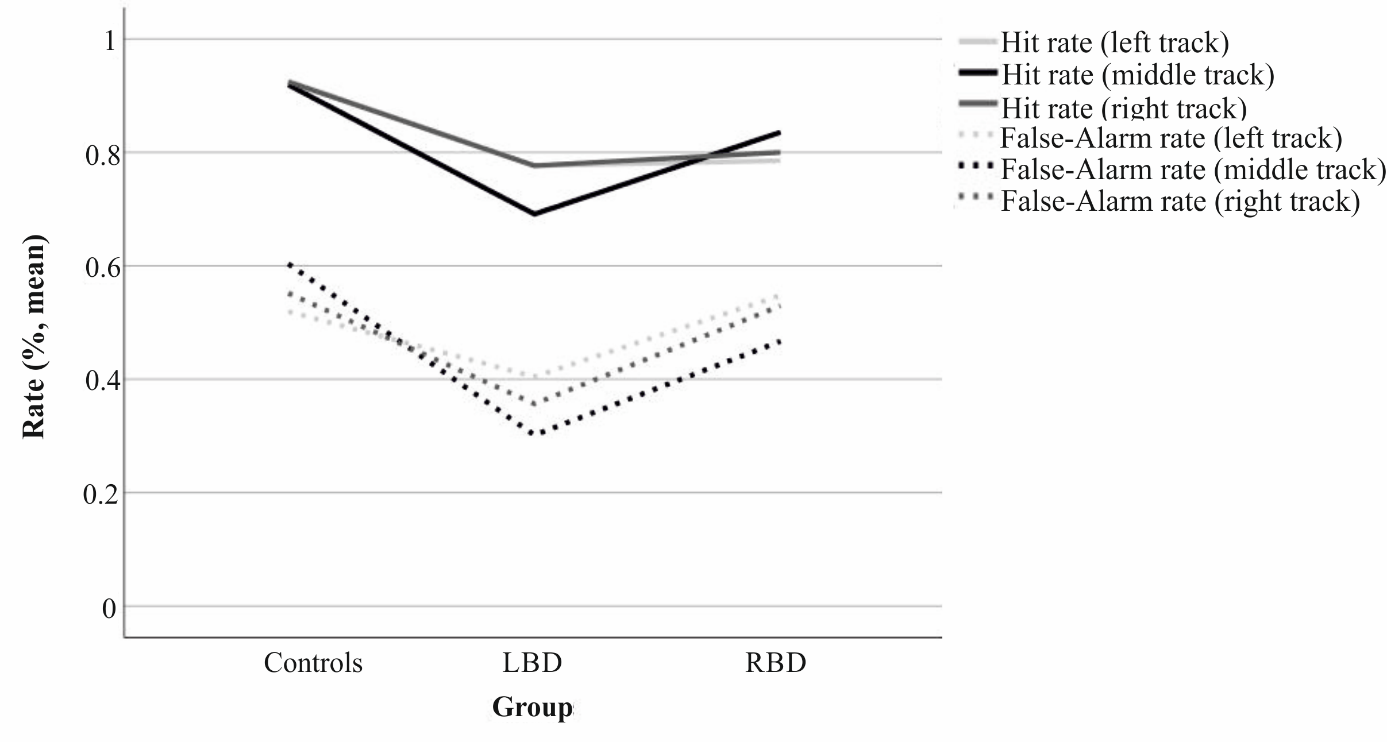


**Supplementary Figure 1. Hit and False Alarm rates per group and track (left, middle, right).**

**Correlations of Neuropsychological Assessment and Reachability Judgments.**

Results of additional and exploratory analyses are depicted below. s. Please note that p-values presented here are not corrected for multiple comparisons.

| \| Supplementary Table 2.  *Correlations between neuropsychological abilities or motor function and reachability judgment performance per patient group.* \|  \| \| --- \| --- \| | | | | | | | |
| --- | --- | --- | --- | --- | --- | --- | --- | --- | --- |
| **Group** | **Neuro-Asessment** | **Accuracy** (%) | **Hit rate** | **FA rate** | **Perceptual sensitivity** (d-prime) | **Judgment tendency** (criterion) | **Perceptual accuracy** (AUC) |
| LBD | Line Bisection | -.295 | -.214 | 0 | **-.415^*^** | .078 | -.221 |
|  |  | (.164) | (.312) | (1) | **(.045)** | (.707) | (.286) |
|  | Star Cancellation | **-.538**** | -.040 | .131 | -.275 | -.059 | **-.482*** |
|  |  | **(.009)** | (.844) | (.523) | (.171) | (.769) | **(.017)** |
|  | WMFT (functional ability) | .241 | -.109 | -.255 | .241 | .211 | .204 |
|  |  | (.194) | (.558) | (.169) | (.185) | (.246) | (.263) |
|  | VATA-M Self  Evaluation | -.279 | -.116 | .108 | **-.457^*^** | -.052 | -.195 |
|  |  | (.132) | (.531) | (.560) | **(.012)** | (.772) | (.282) |
|  | Imitation Hand | .114 | .008 | .089 | -.008 | -.102 | 0 |
|  |  | (.553) | (.966) | (.642) | (.967) | (.585) | (1) |
|  | Pantomime | .101 | .062 | .039 | .105 | -.090 | .015 |
|  |  | (.587) | (.738) | (.835) | (.562) | (.619) | (.934) |
| RBD | Line Bisection | .179 | .245 | .075 | .205 | -.158 | .158 |
|  |  | (.372) | (.243) | (.707) | (.302) | (.426) | (.426) |
|  | Star Cancellation | -.083 | .018 | -.107 | .025 | .041 | -.008 |
|  |  | (.666) | (.929) | (.575) | (.897) | (.830) | (.966) |
|  | WMFT (functional ability) | -.163 | .294 | .240 | -.139 | -.268 | -.146 |
|  |  | (.378) | (.128) | (.194) | (.451) | (.143) | (.427) |
|  | VATA-M Self Evaluation | .336 | **-.396^*^** | **-.479^**^** | .309 | **.466^*^** | **.481^**^** |
|  |  | (.067) | **(.038)** | **(.009)** | (.089) | **(.010)** | **(.008)** |
|  | Imitation Hand | .353 | .056 | -.197 | .366 | .119 | .374 |
|  |  | (.070) | (.784) | (.311) | (.058) | (.538) | (.053) |
|  | Pantomime | -.194 | .101 | .193 | -.176 | -.122 | -.168 |
|  |  | (.296) | (.603) | (.297) | (.338) | (.505) | (.360) |


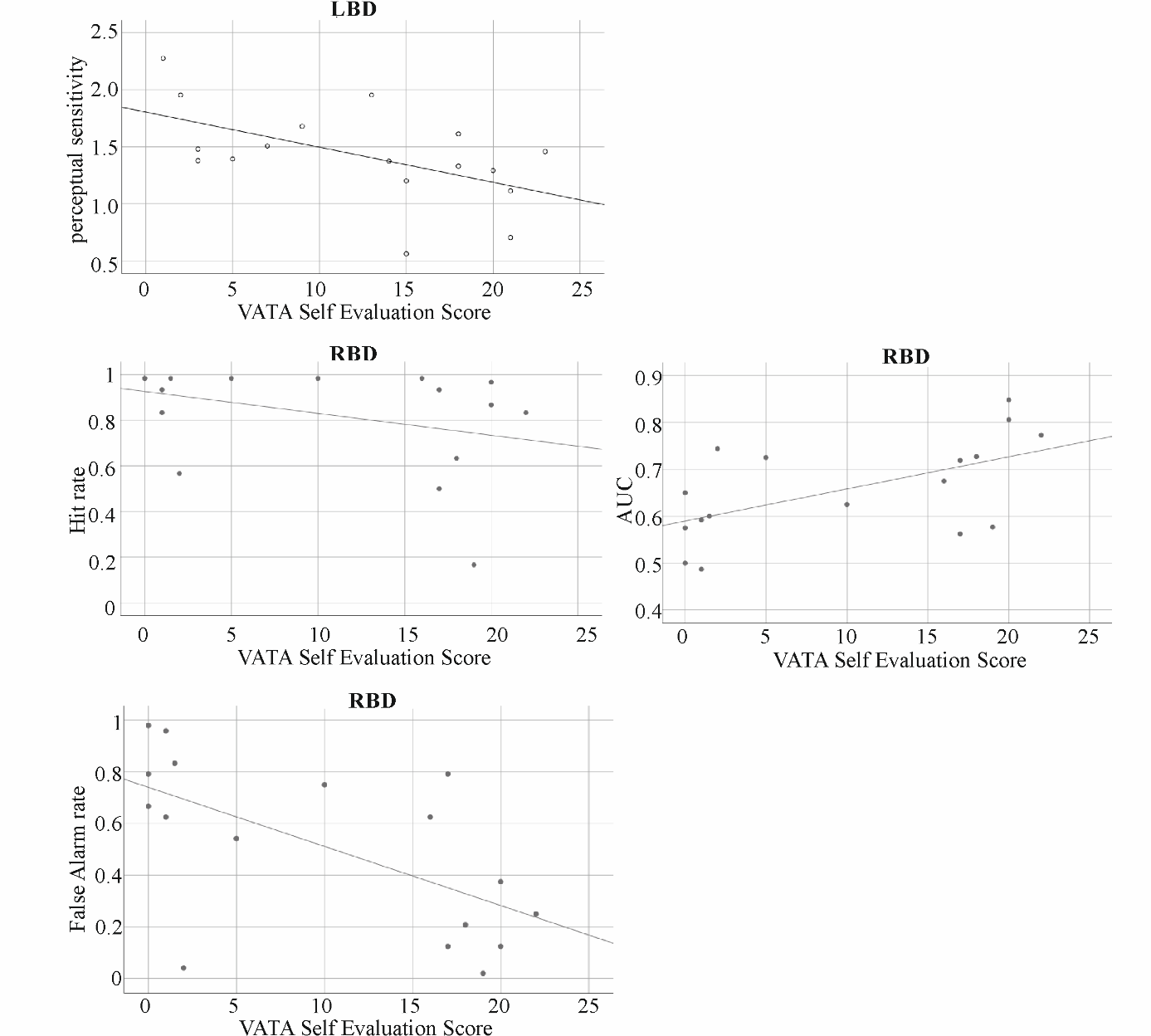


**Supplementary Figure 2. Correlations of Neuropsychological Assessment and Reachability Judgments.** Correlation plots show VATA self-evaluation scores (x-axis) and the respective variable describing reachability judgments (y-axis). Please note that we here only depict those neuropsychological variables that significantly correlated with reachability judgments. Further, the correlation between VATA self-evaluation score and judgment tendency (criterion) in the RBD group is already depicted in Fig. 4 in the main text.
